# Rare pulmonary lymphangioleiomyomatosis complicated with tuberculosis: a Case Report and literature review

**DOI:** 10.3389/fmed.2026.1796995

**Published:** 2026-04-13

**Authors:** Xiangnan Zhang, Luyao Fang, Xinchao Zhang, Qian Du, Shuo Xu, Huanfen Zhao

**Affiliations:** 1 Department of Pathology, Hebei General Hospital, Shijiazhuang, China; 2 Department of Pathology, Affiliated Hospital of Hebei University of Engineering, Handan, China; 3 Department of Nuclear Medicine, Hebei General Hospital, Shijiazhuang, China; 4 Department of Gynecology, Hebei General Hospital, Shijiazhuang, China

**Keywords:** clinical pathology, immunohistochemistry, pathological diagnosis, pulmonary lymphangioleiomyomatosis, tuberculosis

## Abstract

**Background:**

Pulmonary lymphangioleiomyomatosis (PLAM) is a rare progressive interstitial lung disease characterized by diffuse cystic lesions predominantly affecting women of childbearing age. Its clinical manifestations are diverse and nonspecific. Cases of PLAM complicated with pulmonary tuberculosis (TB) are extremely rare. Here, we retrospectively report one case of PLAM with concurrent TB and review the relevant literature to summarize its clinical, imaging, pathological, diagnostic, therapeutic, and prognostic features, with the aim of providing references for the pathological diagnosis and differential diagnosis of PLAM.

**Case presentation:**

A 36-year-old female was admitted with left pneumothorax and left lung nodules. Chest CT demonstrated a solid nodule in the anterior basal segment of the left lower lobe, tiny nodules in the left upper lobe and bilateral lower lobes, bilateral emphysema, left hydropneumothorax, minimal right pleural effusion, and focal right pleural thickening. Cardiopulmonary function tests, including echocardiography, pulmonary function testing, ECG, and blood gas analysis, were unremarkable. The patient underwent “single-port thoracoscopic wedge resection of the left lower lung lobe + pleural adhesion lysis + pleurodesis”. The resected lesion from the left lower lobe was submitted for histopathological examination, which demonstrated epithelioid granulomas with coagulative necrosis, adjacent parenchymal emphysema and bullae, and spindle/epithelioid smooth muscle-like cells proliferation along cyst walls. Immunohistochemistry was positive for SMA, HMB45, Desmin, PR, and BRAF V600E. Special stains were negative for acid-fast bacilli, PAS, and PASM. Molecular testing confirmed a BRAF V600E mutation. A final diagnosis of PLAM complicated with TB was established.

**Conclusion:**

PLAM is a rare lung disease characterized primarily by diffuse interstitial lung lesions. It poses challenges in early diagnosis and has a poor prognosis. Its relationship with TB is complex, as the two conditions mutually promote each other and can act as both cause and effect. When the two coexist, the clinical manifestations are diverse. In-depth exploration of their pathological characteristics, diagnosis, and differential diagnosis is conducive to clinical diagnosis and treatment.

## Introduction

PLAM is a rare interstitial lung disease characterized by diffuse cystic lesions (1). Clinical manifestations are diverse and non-specific, including chest pain, dyspnea, recurrent pneumothorax, chylothorax, retroperitoneal lymphadenopathy, and renal angiomyolipomas. PLAM predominantly affects women of childbearing age, while cases in males and children are extremely rare (2, 3). The coexistence of PLAM and tuberculosis (TB) is even rarer. The two conditions interact reciprocally and promote each other's progression. Here, we report a case of PLAM complicated with TB and review the relevant literature to investigate its clinical, imaging, and pathological features, as well as the interaction between PLAM and TB, aiming to provide a reference for the diagnosis and treatment of this rare comorbidity.

## Case presentation

A 36-year-old female was admitted on June 20, 2023, with sudden-onset left-sided chest pain, chest tightness, and dyspnea lasting for 4 days. Physical examination showed hyperresonance on percussion, slightly reduced vocal fremitus, and diminished breath sounds over the left lung, with no rales auscultated. Physical examinations of the cardiac, abdominal, and nervous systems revealed no abnormalities. The patient was of childbearing age with regular menstrual cycles. She had no history of hormonal drug use, smoking, or alcohol consumption, nor a past medical history of pulmonary diseases. There was no family history of PLAM, pulmonary TB, or other hereditary disorders.

**Figure 1:**
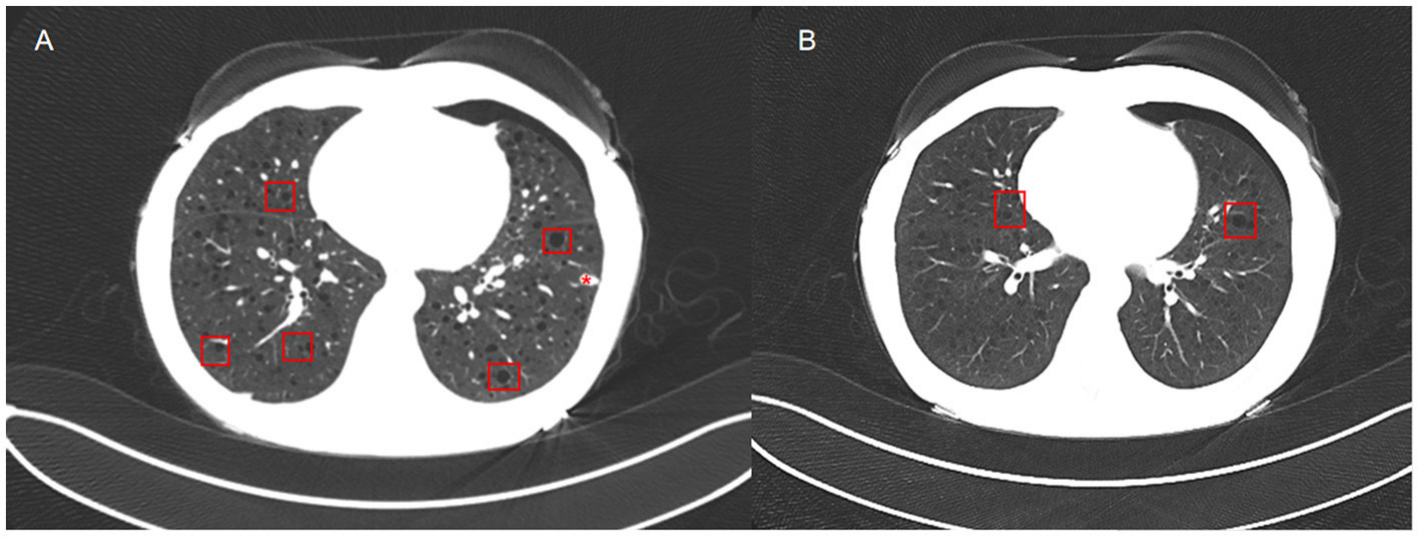
**(A, B)** High-resolution computed tomography (HRCT) shows multiple diffuse round small cystic spaces in both lungs at different levels, with uniform distribution and varying sizes. “□” indicates dilated small cystic spaces, and “*” indicates pulmonary tuberculosis nodules.

Chest HRCT (Figures 1A, B) showed diffuse, uniformly distributed thin-walled small cysts (2–10 mm in diameter) with well-circumscribed margins in both lungs, consistent with early-stage lymphangioleiomyomatosis. A solid nodule was detected in the anterior basal segment of the left lower lobe, accompanied by micronodules in the left upper lobe and bilateral lower lobes. Additional findings included bilateral pulmonary emphysema, left hydropneumothorax, minimal right pleural effusion, and focal right pleural thickening. Cardiopulmonary function tests (echocardiography, pulmonary function testing, electrocardiogram, and blood gas analysis) were unremarkable. Liver and renal function, as well as inflammatory markers (C-reactive protein and procalcitonin), were within normal ranges.

**Figure 2:**
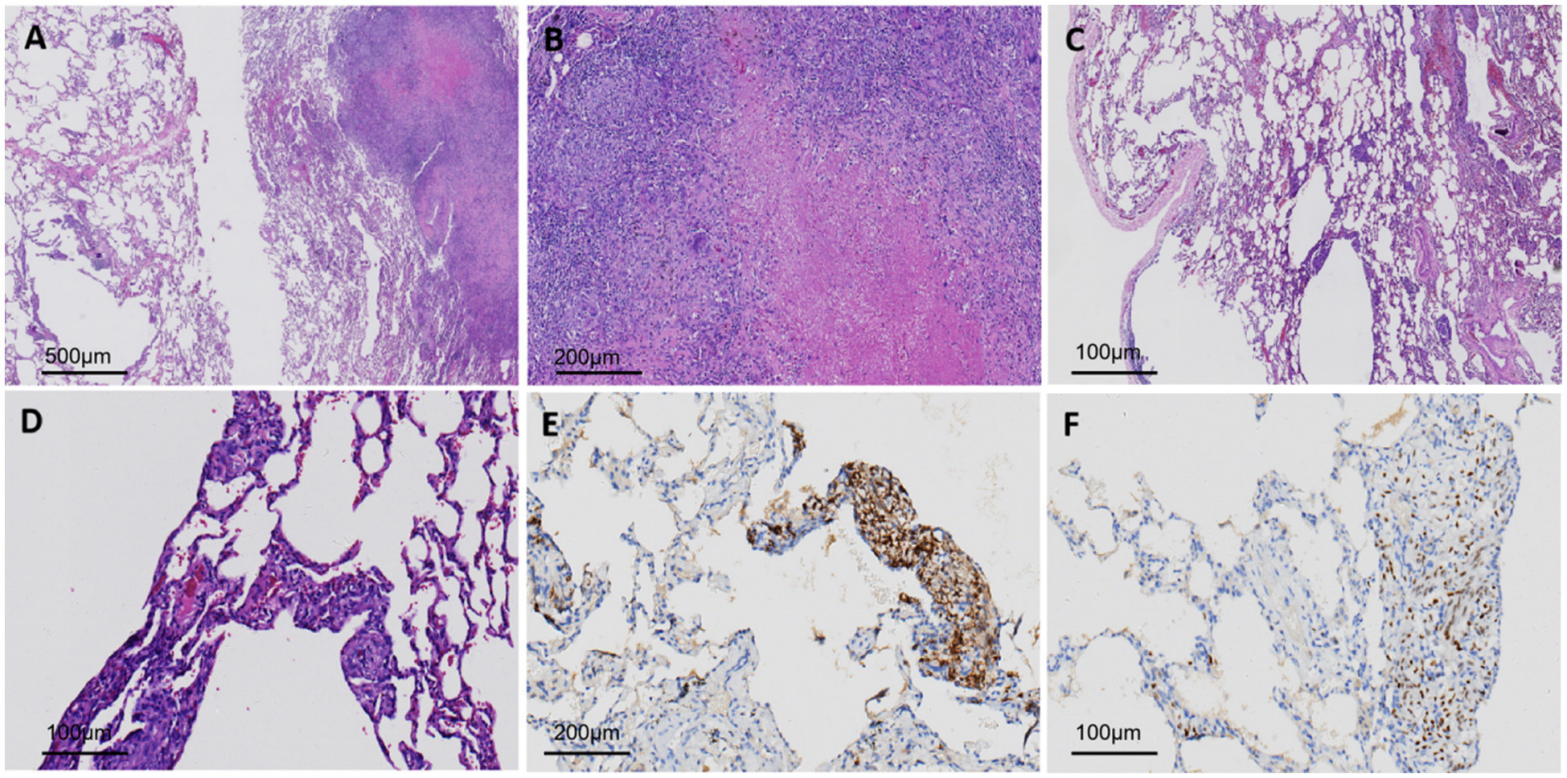
**(A)** Cystic dilatation areas **(left)** and tuberculosis areas **(right)** observed under low magnification, 40 × (**B)** Caseous necrosis and multinucleated giant cell reaction, 200 × **(C)** Multiple cystic dilatation areas, with tumor cells growing along the alveolar walls, 200 × **(D)** Tumor cells are oval or spindle-shaped, with clear and light eosinophilic cytoplasm and short spindle-shaped nuclei without obvious atypia; they grow along blood vessels, lymphatic vessels or bronchioles, and alveolar cystic dilatation is present, 400 × **(E)** Tumor cells are HMB45-positive, 400 × **(F)** Tumor cells are ER-positive, 400 × .

Following anti-infective and nebulized mucolytic treatment, the patient underwent “single-port thoracoscopic wedge resection of the left lower lung lobe + pleural adhesion lysis + pleurodesis” on June 25, 2023. The resected specimen was submitted for histopathological evaluation. Gross examination demonstrated multiple small cysts within the pulmonary parenchyma and a 1 cm solid subpleural nodule located 0.2 cm deep to the pleural surface. Histologically (Figures 2A–F), the cystic regions showed proliferation of spindle/epithelioid smooth muscle-like cells along cyst walls, vessels, lymphatics, and peribronchiolar structures, with eosinophilic cytoplasm and absent significant cytologic atypia. Immunohistochemical profile was as follows: SMA(+), HMB45(+), desmin(+), PR(+), and BRAF V600E(+). The solid nodule exhibited extensive coagulative necrosis accompanied by multinucleated giant cells and epithelioid histiocytes, consistent with tuberculous pathology. Special stains for acid-fast bacilli, PAS, and PASM were all negative. Given the low sensitivity (20%−30%) of acid-fast staining in localized tuberculosis, a negative result did not exclude the diagnosis. Marked eosinophilia in the patient's serum, a strongly positive purified protein derivative (PPD) test, and confirmation by a specialized tuberculosis center further supported the diagnosis of tuberculosis. Molecular analysis confirmed a BRAF V600E mutation. Based on integrated clinical, radiological, histopathological, immunohistochemical, and molecular findings, a final diagnosis of early-stage PLAM coexisting with pulmonary TB was established.

Postoperatively, the patient underwent closed thoracic drainage with successful re-expansion and no surgical sequelae. She was discharged on postoperative day 5 (June 29, 2023) on oral Shengxuebao Mixture (15 ml po tid) and Yiqi Weixue Capsules (four capsules po tid), with instructions for routine follow-up including chest CT and hepatic/renal function tests. The patient declined standard anti-tuberculosis therapy. Notably, the patient presented with early-stage PLAM, manifesting as isolated bilateral diffuse cystic lesions without recurrent pneumothorax, preserved pulmonary function, and normal carbon monoxide diffusion capacity (DLco). No extrapulmonary involvement was identified. In accordance with the 2017 ATS/JRS clinical practice guidelines for PLAM, clinical observation with routine surveillance was selected as the first-line management strategy. Initiation of targeted pharmacotherapy is planned upon objective evidence of disease progression.

At the last follow-up in August 2025 (26 months post-intervention), the patient remained in favorable general condition, with no recurrence of clinical symptoms including pneumothorax, chest pain, dyspnea, fever, or cough, and maintained normal daily activity tolerance. Follow-up chest HRCT showed stable cystic lesions of lymphangioleiomyomatosis in both lungs without progression, no recurrent hydropneumothorax or pleural effusion, and no residual foci of pulmonary tuberculosis.

During follow-up, the patient maintained a good quality of life without impairment of daily activities, work, or social functioning. She was satisfied with the surgery and postoperative treatment, complied strictly with scheduled follow-up, and had a clear understanding of the rarity and potential progressive risk of PLAM. No psychological abnormalities related to the rare disease diagnosis were observed, and her mental status remained stable.

## Discussion

PLAM is a rare pulmonary disorder characterized predominantly by diffuse interstitial lung lesions (1). Accumulating evidence indicates that PLAM, angiomyolipoma (AML), and clear cell sugar tumor of the lung (CCST) all belong to the perivascular epithelioid cell tumor (PEComa) family. PLAM can be classified into two distinct subtypes: sporadic lymphangioleiomyomatosis (S-LAM) and tuberous sclerosis complex-associated lymphangioleiomyomatosis (TSC-LAM).

PLAM exhibits diverse and non-specific clinical manifestations, characterized mainly by chronically progressive, diffuse cystic lesions in both lungs, which may be associated with chest pain, dyspnea, recurrent pneumothorax, chylothorax, retroperitoneal lymphadenopathy, and renal angiomyolipoma. The disease predominantly affects women of childbearing age, with cases in males and children being exceedingly rare (2, 3). The lack of specific clinical features renders the clinical diagnosis of PLAM challenging. Conventional imaging modalities, including chest radiography and standard computed tomography (CT), demonstrate limited diagnostic accuracy for PLAM, whereas high-resolution computed tomography (HRCT) serves as the optimal imaging tool (4). The characteristic radiological findings consist of diffuse, evenly distributed small thin-walled cysts in both lungs, which are round or oval in morphology. The majority of cysts range from 2 to 25 mm in diameter, with a cyst wall thickness typically less than 1 mm and distinct margins.

This case presented substantial diagnostic challenges owing to overlapping clinical and radiological manifestations between PLAM and pulmonary TB. The patient initially presented with left pneumothorax, a shared finding in both entities. Chest HRCT demonstrated localized solid nodules accompanied by multiple cystic lesions, which precluded reliable preoperative differentiation between PLAM and TB lesions. Definitive diagnosis was established via multimodal integrated assessment:Histopathologic examination revealed concomitant characteristic features of PLAM (spindle cell proliferation within cyst walls) and pulmonary TB (epithelioid granulomas with caseous necrosis);Immunohistochemical profiling (positive expression of HMB45, SMA, PR, and BRAF V600E) confirmed the diagnosis of PLAM. Notably, a negative acid-fast stain did not exclude pulmonary TB, attributable to the low detection rate of acid-fast bacilli in localized lesions.

Patients with PLAM frequently show reduced diffusing capacity and obstructive ventilatory dysfunction, with some accompanied by hypoxemia. Pulmonary function tests, serology, and blood gas analysis provide supplementary diagnostic evidence (5). According to the 2017 ATS/JRS clinical practice guidelines, a serum VEGF-D level ≥ 800 pg/ml is characteristic of PLAM (6). VEGF-D correlates with disease severity, chylous effusion, and lymphatic involvement; levels are lower in patients with isolated pulmonary cysts than in those with chylothorax, extrapulmonary involvement, or tuberous sclerosis complex, and lymphatic involvement is more common at VEGF-D ≥ 800 pg/ml (7). As an extracellular matrix remodeling biomarker, MMP2 differentiates PLAM from other cystic lung diseases and healthy controls. Combined measurement of serum MMP2 and VEGF-D improves diagnostic accuracy (8). Moreover, serum VEGF-D serves as a biomarker for predicting the therapeutic response to mTOR inhibitors (9).

The pathogenesis of PLAM remains incompletely elucidated. Current evidence suggests (5):①Mutations in the tuberous sclerosis complex genes TSC1/2 lead to mTOR activation, which promotes abnormal proliferation of vascular smooth muscle and PLAM cells, resulting in lung tissue destruction and remodeling. ②PLAM occurs predominantly in women of childbearing age, and pregnancy, menstruation, estrogen, or contraceptive use may exacerbate the disease. The incidence of PLAM in pregnant women is 11-fold higher than in healthy controls (10), implying a close association with overexpression of estrogen, progesterone, and matrix metalloproteinases (MMP2, MMP5, etc.) (5). ③Abnormal expression of HMGA2 contributes to the pathogenesis of PLAM (11).

The relationship between PLAM and pulmonary TB remains poorly elucidated. This association is complex and supported only by a limited number of clinical cases. A literature search of the PubMed database identified merely 2 relevant reported cases (2, 3). A plausible mechanism is that PLAM-induced lung parenchymal structural damage (e.g., diffuse cysts, bullae) impairs the pulmonary physical barrier function, thereby increasing susceptibility to Mycobacterium tuberculosis colonization and infection (3). Notably, one of the reported patients developed secondary pulmonary tuberculosis 2 months after initiating sirolimus therapy, which may be attributed to the immunosuppressive effects of sirolimus. In the present case, the patient presented primarily with pneumothorax and exhibited typical radiological features of PLAM, while the tuberculous lesion was incidentally detected during the current admission. Beyond PLAM-related lung structural damage, the patient had no history of tuberculosis exposure or other infection risk factors, nor had they received sirolimus treatment. We therefore hypothesize that PLAM-induced parenchymal structural alterations facilitated Mycobacterium tuberculosis infection, further confirming that PLAM itself—independent of immunosuppressive agents—can augment the risk of pulmonary tuberculosis by compromising the pulmonary barrier function. This finding underscores the importance for clinicians to monitor the risk of pulmonary tuberculosis and other infectious diseases even in patients with early-stage PLAM who have not undergone immunosuppressive therapy. Timely diagnostic evaluations should be performed when suspicious symptoms arise.

Pathological diagnosis represents the gold standard for PLAM. Grossly, the lungs exhibit multiple cystic lesions with walls slightly thicker than those observed in emphysema and a soft consistency (4). Microscopically, abnormal proliferation of spindle-shaped or epithelioid smooth muscle cells is present within the pulmonary interstitium, surrounding lymphatics, small blood vessels, and small airways and leading to luminal stenosis and obliteration. These changes result in distal bronchial air trapping, obstruction or rupture of pulmonary venules, and impaired lymphatic drainage, which ultimately give rise to pulmonary bullae, recurrent spontaneous pneumothorax, hemoptysis, and chylothorax (12). Immunohistochemically, tumor cells are positive for HMB45, SMA, desmin, ER, and PR, with HMB45 demonstrating the highest diagnostic significance (13, 14). PLAM may also highly specifically express β-catenin, which can be utilized as an auxiliary diagnostic marker.

PLAM is easily misdiagnosed as other diseases characterized by multiple pulmonary cystic lesions (15), necessitating careful differential diagnosis. ①Pulmonary Langerhans cell histiocytosis (PLCH): predominantly affects young males with a history of smoking, with lesions mainly distributed in the upper and middle lung zones. Microscopically, nodular proliferation of lesional cells with eosinophilic granulomas is seen; the cells exhibit abundant cytoplasm, convoluted nuclei, and nuclear grooves. Immunohistochemically, the cells are positive for CD1α, Langerin, and S-100. ②Lymphocytic interstitial pneumonia (LIP): associated with autoimmune diseases. Although cystic changes may be present, marked infiltration of inflammatory cells and histiocytes is observed in the alveolar septa.③Birt-Hogg-Dubé (BHD) syndrome: a rare autosomal dominant monogenic disorder with clinical and radiological features mimicking PLAM. Diagnosis can be confirmed by family history evaluation and FLCN gene mutation analysis. ④Cystic bronchiectasis: cystic spaces are distributed along the bronchial tree with relatively thick walls. Pediatric ⑤PLAM should be differentiated from congenital pulmonary cysts: chest CT demonstrates well-circumscribed thin-walled round or oval cysts (air-filled, fluid-filled, or air-fluid level), often associated with pulmonary hypoplasia; no carbon deposition is identified microscopically.

Patients with PLAM often develop respiratory failure or death within 10 years following symptom onset (16). As the pathogenesis remains incompletely understood, current therapeutic strategies are limited in efficacy, and clinical management mainly consists of pharmacologic and surgical interventions (17). ①Pharmacologic therapy. First-line medications include mTOR inhibitors (e.g., sirolimus), statins, hydroxychloroquine, cyclooxygenase inhibitors, tyrosine kinase inhibitors, bronchodilators, and immunosuppressants (18). Additional options comprise doxycycline (a matrix metalloproteinase inhibitor), anti-estrogen/progesterone therapy, and anti-VEGF-D therapy (19). Sirolimus is the first-line agent; early and long-term use stabilizes pulmonary function and improves quality of life (20). Studies have demonstrated that sirolimus reduces the recurrence rate of pneumothorax or chylothorax (1), supporting its early use in patients with initial pneumothorax or chylothorax. Sirolimus is also indicated in patients with extrapulmonary involvement (12). Traditional Chinese medicine may provide additional therapeutic benefits. ②Surgical therapy. Video-assisted thoracoscopic surgery (VATS) with wedge resection is indicated for PLAM patients with pneumothorax. Pleurodesis via pleural abrasion and talc poudrage yields satisfactory outcomes. Closed thoracic drainage is an effective alternative for inoperable patients. ③Lung transplantation. Lung transplantation is a viable option for patients with end-stage PLAM (21). Among 57 transplant recipients, the 1-year, 3-year, 5-year, and 10-year survival rates were 86.7, 82.5, 73.7, and 73.7%, respectively (22). However, disease recurrence may occur post-transplantation. ④Potential therapeutic targets. Targeting the SPHK1/S1P/S1PR3 signaling pathway may represent a promising therapeutic strategy for PLAM (23), offering novel directions for future research.

This study has several limitations. First, as a single-case report, the findings require validation through multicenter, large-sample clinical studies to further confirm the association between the two conditions and refine diagnostic and therapeutic strategies. Second, the follow-up duration (26 months) was relatively short; long-term follow-up (≥5 years) is warranted to monitor the long-term progression of pulmonary lymphangioleiomyomatosis (PLAM), the risk of pulmonary tuberculosis recurrence, and the therapeutic efficacy of sirolimus or BRAF inhibitors that may be initiated in the future. Third, serum levels of VEGF-D and MMP2—key biomarkers for assessing PLAM severity and disease progression—were not measured, which constrained the comprehensive evaluation of the patient's condition. These biomarkers have now been incorporated into the patient's routine follow-up monitoring protocol.

## Conclusion

Early-stage PLAM is prone to misdiagnosis. When a patient experiences recurrent pneumothorax, the possibility of PLAM should be considered, and a pathological biopsy should be performed. Cases of PLAM complicated with pulmonary tuberculosis are extremely rare, but such comorbidity makes treatment more difficult and significantly impairs the patient's quality of life. On one hand, long-term chronic damage to the lung parenchyma leads to a decline in lung resistance; on the other hand, the immunosuppressive effect of sirolimus creates an opportunity for opportunistic pathogens to cause infection. This reminds us that in the treatment of PLAM, greater attention should be paid to the risk of concurrent infection with Mycobacterium tuberculosis and other pathogens, and more caution should be exercised when administering medications, particularly regarding dosage and drug-drug interactions.

## Data Availability

The original contributions presented in the study are included in the article/supplementary material, further inquiries can be directed to the corresponding author.
